# Supplementary material for: The interactome of intact mitochondria by cross-linking mass spectrometry provides evidence for coexisting respiratory supercomplexes
Source: Mol Cell Proteomics. 2017 Dec 8;17(2):216–32. doi: 10.1074/mcp.RA117.000470 (PMC5795388; doi:10.1074/mcp.RA117.000470)
Supplement: Supplemental Data [file supp_RA117.000470_133922_0_supp_23978_fzffwf.zip › spectra_annotation/mito_spectra_annotation/248-1-5-1-6-1.pdf]

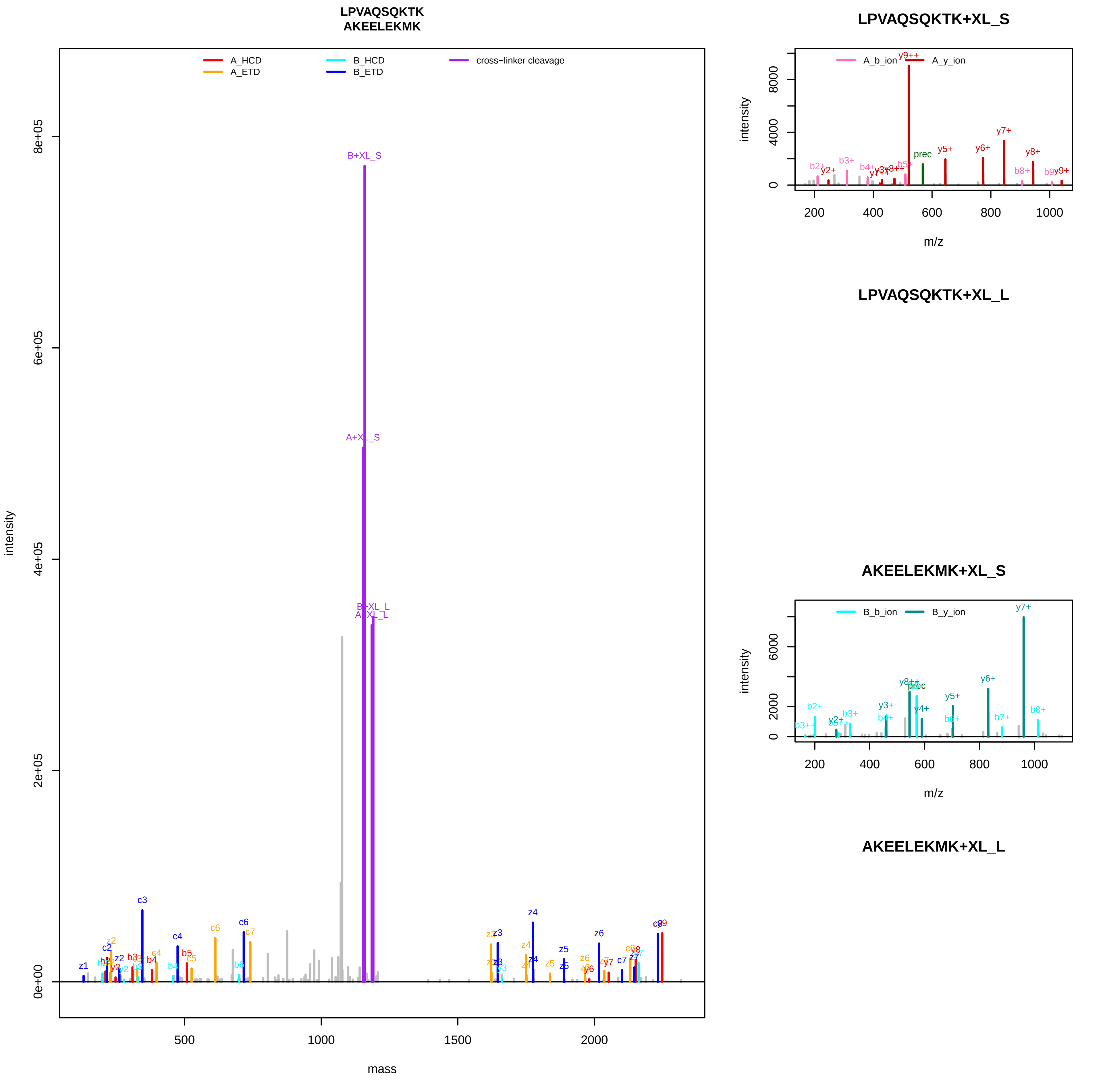
LPVAQSQKTK
AKEELEKMK
A_HCD
A_ETD
B_HCD
B_ETD
cross−linker cleavage
B+XL_S
A+XL_S
B+XL_L
A+XL_L
intensity
mass
0e+00
2e+05
4e+05
6e+05
8e+05
500
1000
1500
2000
z1
c2
z2
b3
c3
b4
c4
b5
c5
c6
c7
z3
z4
z5
z6
y6
y7
c7
c9
LPVAQSQKTK+XL_S
A_b_ion
A_y_ion
y9++
prec
y2+
b2+
b3+
b4+
b5+
y5+
y6+
y7+
y8+
b8+
y9+
m/z
0
4000
8000
200
400
600
800
1000
LPVAQSQKTK+XL_L
AKEELEKMK+XL_S
B_b_ion
B_y_ion
y7+
y8++
y6+
y5+
y4+
y3+
b4+
b2+
b3+
y2+
b6+
b7+
b8+
0
2000
6000
AKEELEKMK+XL_L
